# Targeting Rap1-YAP1 mechanosignaling for ameliorating acute IOP elevation-induced trabecular meshwork dysfunction

**DOI:** 10.1016/j.isci.2026.116268

**Published:** 2026-06-05

**Authors:** Yupeng Zhang, Xue Li, Qiumei Hu, Linlin Luo, Qian Luo, Xiangbin Guan, Jingyi Zhu

**Affiliations:** 1 Department of Ophthalmology, The General Hospital of Central Theater Command, Wuhan, Hubei Province 430000, China; 2 School of Life Sciences, Tsinghua University, Beijing 100084, China; 3 Department of Ophthalmology, Institute of Surgery Research, Army Medical Center of PLA (Daping Hospital), Army Medical University, Chongqing 400042, China; 4 Wuhan University of Science and Technology, Wuhan, Hubei Province 430065, China

**Keywords:** Molecular biology, Ophthalmology

## Abstract

Glaucoma is the second leading cause of blindness, and intraocular pressure (IOP) is the principal and only modifiable risk factor. The trabecular meshwork (TM) remains the most important target for IOP regulation; although it is highly mechanosensitive, TM responses to high IOP remain unclear. This study suggested that human TM cells are susceptible to mechanical stimuli using single-cell sequencing; further, *in vivo* and *in vitro* models of acute ocular hypertension were established. TM cells underwent significant cell death and cytoskeletal rearrangement after acute IOP elevation. Expression of classic fibrotic biomarkers was significantly upregulated in both mice and human TM cells, whereas levels of phosphorylated YAP1 and Rap1 were reduced. Notably, Rap1b knockin mice exhibited no significant TM death, extracellular matrix disruption, or upregulation of YAP1 following acute IOP elevation. Accordingly, Rap1-YAP1 mechanosignaling might be a novel therapeutic target for TM protection following glaucoma-associated acute IOP elevation.

## Highlights

• HTM cells are susceptible to mechanical stimuli based on scRNA-seq analysis
• TM undergoes significant cell death and cytoskeletal rearrangement after acute HIOP
• Rap1-YAP1 mechanosignaling regulates TM following acute HIOP

## Graphical abstract

**Figure:**
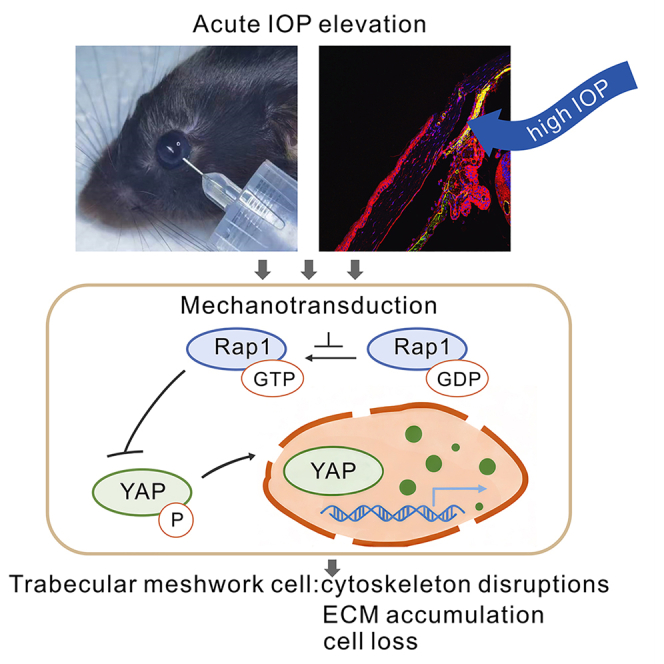

## Introduction

The trabecular meshwork (TM), which is the principal site of aqueous humor (AH) outflow resistance in the conventional outflow pathway, is responsible for approximately 85% of the AH outflow under physiological conditions.^1^ Pathological changes in the TM, such as excessive deposition of extracellular matrix (ECM) and abnormal cytoskeletal rearrangement, are the main contributors to increase AH outflow resistance, resulting in high intraocular pressure (HIOP), which is a major risk factor for glaucomatous neurodegeneration.^2^^,^^3^^,^^4^ A better understanding of the pathophysiological mechanisms of the TM is critical for the development of innovative IOP-lowering strategies for treating glaucoma.

The TM is a highly fenestrated lattice-like biological filter comprising several irregular ECM layers lined by TM beam cells, extending the entire length of the Schlemm’s canal (SC).^5^ Anatomically, the TM is divided into three filtering regions, through which the AH traverses from the innermost to the outermost: first, the uveal meshwork, then the corneoscleral meshwork, and finally, the juxtacanalicular tissue (JCT).^6^^,^^7^^,^^8^ The uveal and corneoscleral meshworks consist of fenestrated lamellae and large intertrabecular spaces lined by TM cells. The JCT contains TM cells embedded within a loose ECM.^1^^,^^9^ Cell culture of isolated TM cells from human TM tissue has shown that TM cells exhibit heterogeneous phenotypes ranging from endothelial-like to fibroblast-like, myofibroblast-like, and macrophage-like morphologies.^9^ This phenotypic diversity is context-dependent and variable, with mechanical stress and cytokine exposure promoting myofibroblastic transition associated with pathological remodeling.^9^^,^^10^^,^^11^

The TM serves as a biological filter that provides resistance to AH outflow into the SC, with the filtering capacity regulated by various factors. Pathological changes such as ECM accumulation, cytoskeletal disruptions, and cell death can cause tissue stiffness, increasing the resistance to AH outflow.^12^^,^^13^

The TM is continuously exposed to mechanical stimulation, such as with blinking and rhythmic IOP fluctuations that cause morphological deformations. Under physiological conditions, the collagen fibers of the TM are arranged regularly to maintain physiological permeability of the outflow channel. In response to mechanical stretch or strain, mechanosensitive ion channels that are abundantly expressed in the TM, such as Piezo1 and TRPV4, are activated.^14^^,^^15^^,^^16^ Intracellular signaling pathways, including AKT and caveolin-1-mediated signaling, are subsequently activated, leading to changes in the ECM and in cytoskeletal remodeling, cell contractile properties, cytokine secretion, and autophagy.^17^^,^^18^ In this way, the AH outflow can match AH production and maintain the mechanical microenvironment for the retina and optic nerve. However, pathological mechanical stimulation could potentially trigger maladaptive remodeling of the TM, characterized by excessive ECM deposition, disrupted cytoskeleton regulation, and prominent cell death, thereby increasing the resistance to AH drainage.^19^^,^^20^

Although previous work has explored how elevated IOP and mechanical stretch influence TM to some extent, the specific cellular and molecular responses to sudden acute IOP elevation remain incompletely characterized. It is well-established that TM cells are mechanosensitive, and mechanical stimuli such as sustained stretch and elevated IOP can alter gene expression in both normal and pathophysiological conditions.^21^^,^^22^ ECM-related genes and pathways, including transforming growth factor β signaling, are also involved in the responses of TM cells to sustained stretch and elevated IOP.^23^^,^^24^^,^^25^ Understanding the mechanisms associated with ocular hypertension may provide novel insights into the prevention and treatment of glaucoma.^10^

This study aimed to confirm whether human TM cells are susceptible to mechanical stimuli using single-cell RNA sequencing (scRNA-seq) data from human TM samples. Pathological alterations of the TM in response to glaucoma-associated acute IOP elevation were investigated in mouse and human TM cells, and the mechanosensory and downstream signaling pathways that regulate TM dysfunction were explored. Our previous study suggested that the Ras-association proximate 1 (Rap1) pathway is a crucial mediator of pathological changes in the TM in open-angle glaucoma.^26^ Rap1, a small GTPase of the Ras superfamily, acts as a molecular switch that regulates various cellular functions by cycling between the GTP-bound active and guanosine diphosphate (GDP)-bound inactive states.^27^ Rap1 is mechanically activated by shear stress and controls the formation of the endothelial mechanosensing complex and endothelial homeostasis.^28^^,^^29^^,^^30^ Thus, we hypothesized that the Rap1 pathway exerts a significant influence on acute ocular hypertensive phenotypes in the TM and that activating Rap1 signaling could reverse TM dysfunction by inhibiting the Yes-associated protein 1 (YAP1, UniProt: P46937) pathway.

## Results

### scRNA-seq analysis of human TM samples

**Figure 1:**
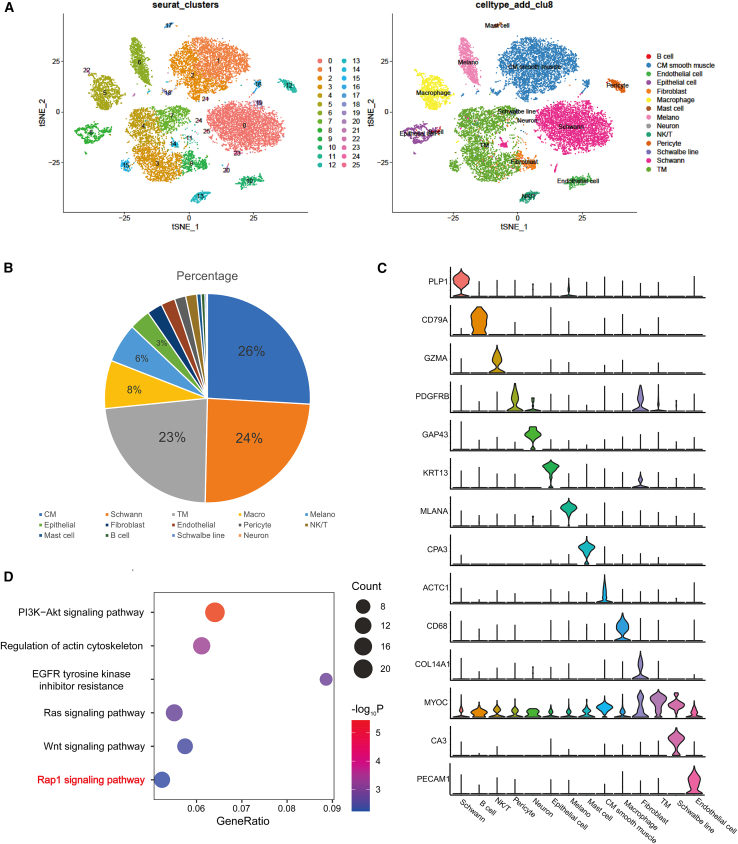
scRNA-seq identification of cell types populating the limbal niche in human (A) Graph-based clustering was performed to group cells according to their gene-expression profile and t-distributed stochastic neighbor embedding (t-SNE) plots were used for visualization. Principal-component analysis of 8,923 single-cell expression profiles obtained from six human trabecular meshwork (TM) samples and one corneoscleral rim. Data are shown in two dimensions using t-SNE. Unsupervised analysis clustered cells into 25 transcriptionally distinct cell populations, each plotted in a different color. (B) The total frequency of each cell type obtained from all samples. (C) Violin plots showing relative expression of the characteristic cell type markers. Columns represent the cell types, while rows represent the marker gene specific to a particular cell type. (D) Enriched Kyoto Encyclopedia of Genes and Genomes pathway of TM cluster are shown.

Single-cell transcriptomes of six human trabecular meshwork (HTM) samples and one corneoscleral rim were subjected to unsupervized clustering. Initial clustering performed at low resolution revealed 25 distinct cell groups (Figure 1A). Differentially expressed genes (DEGs) between clusters were used to identify cell types. Using canonical marker genes, 14 clusters were ultimately defined (Figure 1A), which were visualized by t-distributed stochastic neighbor embedding (t-SNE), ranging from 0.11% to 25.9% in frequency (Figure 1B). The representative marker genes of these clusters are presented as violin plots (Figure 1C). Specifically, Kyoto Encyclopedia of Genes and Genomes (KEGG) analysis indicated that pathway such as regulation of actin cytoskeleton and Rap1 signaling were significantly enriched in TM cells, indicating the potential importance of theses pathways in the pathophysiology of TM (Figure 1D).

**Figure 2:**
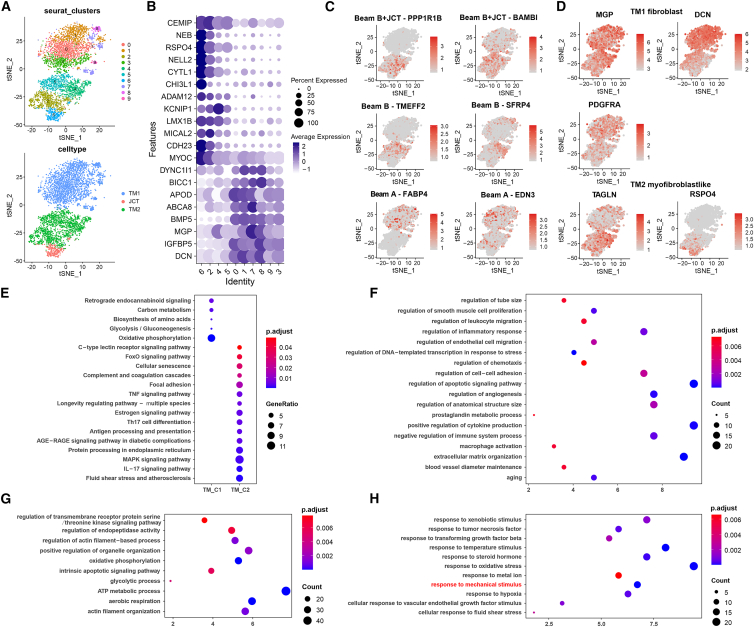
Enriched pathways in trabecular meshwork cell populations (A) t-distributed stochastic neighbor embedding (t-SNE) plots showing previously defined trabecular meshwork (TM) cell populations labeled as three subclusters by the cell population-specific signature genes. (B) Dot plot showing genes selectively expressed in cells of the TM tissue. The size of the dots reflects the proportion of cells of each cell type expressing the marker gene and the intensity of color reflects the mean expression of each marker gene across all the cells. (C) t-SNE plots showing expression of selected marker genes depicting Beam A, Beam B, and juxtacanalicular tissue. Scaled expression levels for each cell were color-coded and overlaid onto the t-SNE plot. (D) t-SNE plots showing expression of selected marker genes depicting fibroblasts and myofibroblasts. (E) Kyoto Encyclopedia of Genes and Genomes pathway enrichment analysis of TM1 and TM2 are shown. (F) Gene Ontology (GO) analysis of TM1 are shown. (G) Gene Ontology (GO) analysis of TM2 are shown. (H) The identified pathways that may affect TM1 from GO analysis.

TM cells, which are a major component of the conventional pathway, were further investigated. Using previously defined TM cell populations, three subclusters based on cell population-specific signature genes were revealed (Figure 2A). A previous study characterized TM cells into three types: Beam A, Beam B, and JCT cells.^6^ Correspondingly in the present study, clusters 3, 9, 8, 7, 1, and 0 exhibited high expression levels of *DCN*, *IGFBP5*, *MGP*, *BMP5*, *ABCA8*, *APOD*, *BICC1*, and *DYNC1I1*, which have been reportedly associated with Beam A cells (Figure 2B).^6^^,^^31^^,^^32^ Beam B cells (clusters 5 and 4) were defined by the expression of *MYOC*, *CDH23*, *MICAL2*, *LMX1B*, *KCNIP1*, and *ADAM12*. The JCT cells (clusters 2 and 6) were defined by the expression of *CHI3L1*, *CYTL1*, *NELL2*, *RSPO4*, *NEB*, and *CEMIP*. The corresponding cell-type markers were also visualized over the t-SNE plots (Figure 2C). The t-SNE plots also revealed that the expression of fibroblast-related marker genes was higher in TM1 than in TM2 and JCT cells, whereas the expression of marker genes associated with myofibroblast-like cells was more prominent in TM2 and JCT than in TM1 cells (Figure 2D).^33^

KEGG analysis was conducted to determine the pathways associated with the TM clusters (Figure 2E). Pathways involving multiple biological processes, such as cellular senescence, the tumor necrosis factor signaling pathway, fluid shear stress, and atherosclerosis, were enriched in the TM1 cluster. However, pathways enriched in the TM2 and JCT clusters mainly related to metabolism, such as glycolysis/gluconeogenesis; Gene Ontology (GO) analysis also supported these results (Figures 2F and 2G). GO analysis (gene >5, p.adjust <0.01) of the TM1 cluster identified gene enrichment in pathways such as ECM organization, aging, and regulation of the apoptotic signaling pathway (Figure 2F), indicating that TM1 cluster cells play central roles as receptors, responding to multiple factors, including oxidative stress, glucocorticoids, hypoxia, and transforming growth factor β, which are well-known factors associated with glaucoma (Figure 2H). Notably, GO analysis revealed that mechanical stimulus response pathways were significantly enriched in the TM1 cluster, suggesting that a portion of TM cells may possess certain mechanosensitive properties (Figure 2H).

### Structural evaluation of the mouse TM following acute IOP elevation

**Figure 3:**
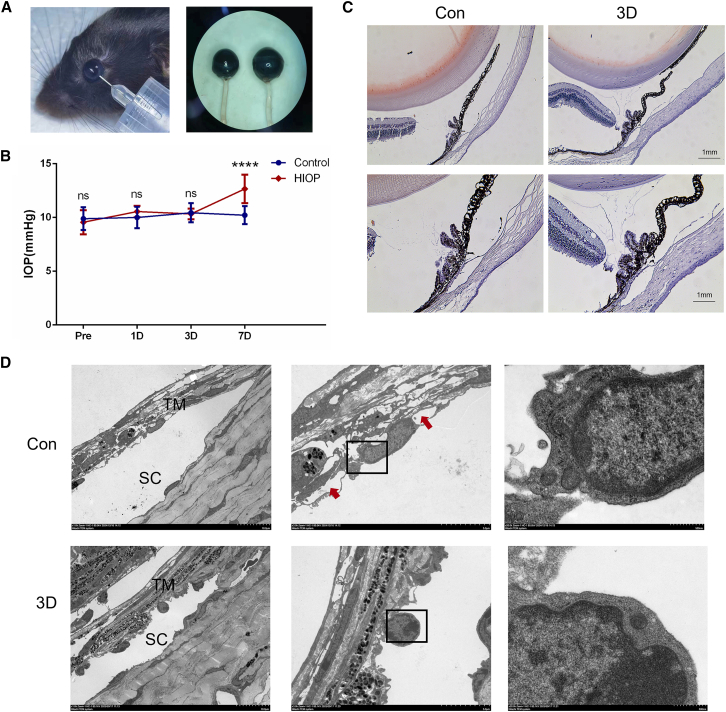
Structural evaluation of the mouse TM following acute IOP elevation (A) Photographs reveal that mice underwent direct cannulation of the anterior chamber (AC) in the eye. Eyeballs were harvested immediately after the procedure, and the gross appearance is illustrated in the right. (B) Intraocular pressure (IOP) measurement following acute ocular hypertension. IOP was measured at baseline (0 days), 3 days, and 7 days following 45 mmHg pressure for 1 h. Data represent mean ± SD. two-way ANOVA was used for comparing IOP between control and HIOP groups across time points. Post hoc test: ns, not significant, ∗*p* < 0.05, ∗∗*p* < 0.01, ∗∗∗*p* < 0.001 vs. control group at corresponding time point. *N* = 9 mice per group. (C) Representative hematoxylin staining of anterior chamber angles 3 days after IOP elevation. Magnification bars: 1 mm. (D) Ultrastructural appearance of the TM tissues by transmission electron microscopy. Leftmost: representative lower magnification image of the TM and SC. Right: higher magnification of the TM cells. The regions encased in black boxes are magnified. TM, trabecular meshwork; SC, Schlemm’s canal; red arrow indicates giant vacuoles.

To further explore how acute HIOP affects the TM, ocular hypertension (45 mmHg for 60 min) was induced in one eye of C57BL/6J mice (Figure 3A). The treated eye was significantly larger than that of the contralateral eye after direct cannulation of the anterior chamber (AC), indicating the successful induction of elevated IOP (Figure 3A). The IOP of both eyes demonstrated gradual increases in the eyes subjected to HIOP 7 days after cannulation (Figure 3B). We next investigated if the structure of the TM or the SC is disrupted, which could lead to increased AH outflow resistance and IOP. The gross morphology of the TM and the SC was observed using hematoxylin staining of eyeball slices; no significant structural disruptions in SC or TM were observed in HIOP or control eyes (Figure 3C). Based on the observations using transmission electron microscopy (TEM), control mouse TMs had a looser structure than those in the HIOP group. Giant vacuoles were frequently observed in the TM of control eyes but were sparse in the HIOP group. The density of the mitochondrial cristae was also reduced in the HIOP TM cells, indicating TM cell damage (Figure 3D). While gross collapse of the SC lumen was not observed, subtle ultrastructural alterations in the inner wall cells were evident, including mitochondrial damage.

### Cellular and cytoskeletal alterations in mouse TM after acute HIOP

**Figure 4:**
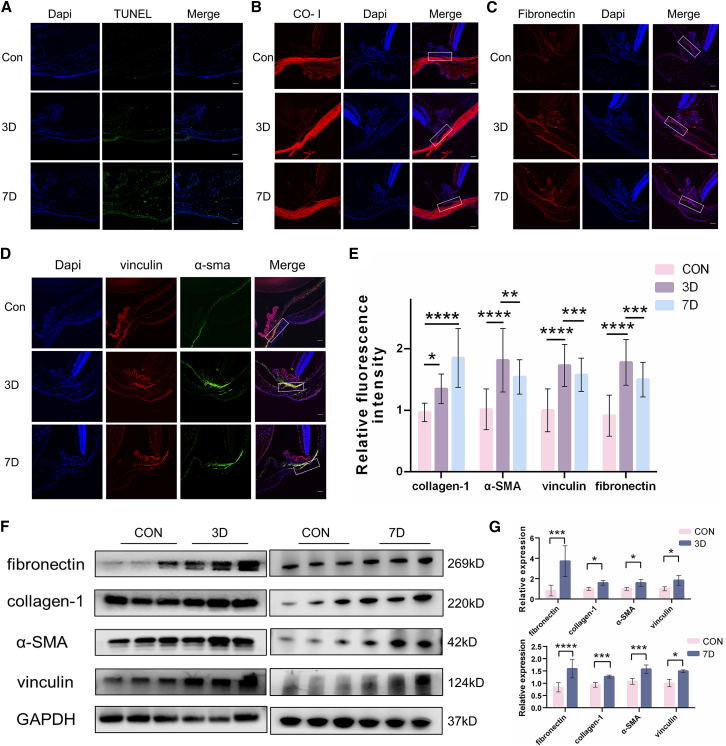
Cellular and cytoskeletal alterations in mouse TM after acute HIOP (A) The anterior chamber angle was stained with cell death marker TUNEL (green) in control eyes (Con), and 3 days (3D), and 7 days (7D) following the acute IOP elevation. DAPI (blue) was used to stain cell nuclei. Magnification bars, 50 μm. (B) The tissue was co-stained with collagen I (red) and DAPI (blue). Magnification bars, 50 μm. (C) The tissue was co-stained with fibronectin (red) and DAPI (blue). Magnification bars, 50 μm. (D) The tissue was co-stained with alpha-smooth muscle actin (α-SMA) (green), vinculin (red) and DAPI (blue). Magnification bars, 50 μm. (E) Quantitative analysis for all immunofluorescence data in Figures 4B–4D. ∗*p* < 0.05, ∗∗*p* < 0.01, ∗∗∗*p* < 0.001, ∗∗∗∗*p* < 0.0001. *N* = 6 mice per group. Data represent mean ± SD. (F) Western blotting showing the protein expression levels of fibronectin, collagen I, α-SMA, and vinculin in the trabecular meshwork tissue from the eyes underwent IOP elevation (HIOP) and the control eyes (CON). (G) Densitometric quantification of fibronectin, collagen I, α-SMA, and vinculin relative to GAPDH protein level in Figure 4F. *N* = 6 mice per group. ns, not significant, ∗*p* < 0.05, ∗∗*p* < 0.01, ∗∗∗*p* < 0.001, ∗∗∗∗*p* < 0.0001. Data represent mean ± SD.

The terminal deoxynucleotidyl transferase-mediated dUTP nick end labeling (TUNEL) assay is designed for detecting cell death, and was performed. Three days after HIOP induction, TUNEL-positive cells were observed around the iris and AC angle and became more common 7 days after HIOP induction, suggesting progressive cell apoptosis (Figure 4A). Fibrosis, which is a common pathological change observed in the glaucomatous TM, is responsible for a stiffened TM and blocked fluid outflow.^34^ Immunolabeling demonstrated that the expression of the collagen-1 and of fibronectin was significantly higher in the HIOP group than in the control group 3 and 7 days after acute HIOP (Figures 4B and 4C). The levels of classic fibrosis biomarker alpha-smooth muscle actin (α-SMA) and vinculin were also greatly increased in HIOP eyes, suggesting active ECM remodeling and increased collagen deposition (Figures 4D and 4E). Western blot analysis confirmed the same trends in the expression levels of collagen-1, vinculin, α-SMA, and fibronectin (Figures 4F and 4G).

### Pathological changes in HTM cells under high hydrostatic pressure

**Figure 5:**
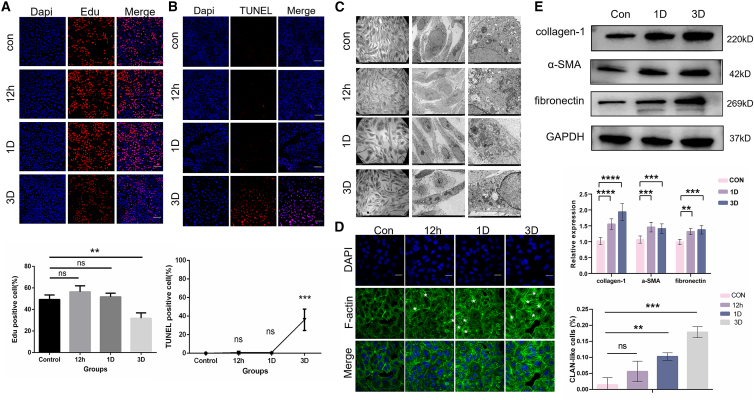
Pathological changes in HTM cells under high hydrostatic pressure (A and B) Representative examples of human trabecular meshwork (HTM) cells cultured under control (con) or high hydrostatic pressure for 12 h, 1 day (1 D), and 3 days (3 D) immunolabeled for EDU (A) and TUNEL (B) assay. Scale bars, 50 μm. Bar graphs summarizing the effects of high hydrostatic pressure on the proliferation and death of HTM cells. *N* = 3. Magnification bars, 100 μm. (C) Histological evaluation of different groups of HTM cells by transmission electron microscopy. *N* = 3. Magnification bars, 50, 10, and 2 μm from the left to the right. (D) Representative fluorescence micrographs of F-actin in HTMs subjected to 12 h, 1D, and 3D culture under high hydrostatic pressure and the control, with nuclei and F-actin labeling shown in blue and green, respectively. The asterisk indicates cross-linked actin network (CLAN+) cells and the percentage of CLAN+ cells was analyzed. *N* = 5. Magnification bars, 25 μm. (E) Expression of fibronectin, collagen I and α-SMA in the different groups of HTM cells by western blots. *N* = 6. ns, not significant, ∗*p* < 0.05, ∗∗*p* < 0.01, ∗∗∗*p* < 0.001, ∗∗∗∗*p* < 0.0001. Data represent mean ± SD.

HTM cells were cultured under high hydrostatic pressure (1,000 Pa) for 12 h, 1 day, or 3 days to mimic ocular hypertension *in vitro*. The 5-ethynyl-2′-deoxyuridine (EDU) and TUNEL assays were performed to examine cell proliferation and death, respectively. Quantitative image analysis revealed that the number of EDU-positive cells significantly decreased after culturing under high hydrostatic pressure for 3 days (Figure 5A). TUNEL-positive nuclei were rarely observed in the 12 h and 1-day groups but were common in the 3-day group (Figure 5B). Ultrastructural analysis revealed enlarged and irregular mitochondria exhibiting membrane damage, matrix dissolution, and loss of cristae in the 3-day group than in the control group (Figure 5C). Subsequently, the TM stiffness was evaluated by detecting ECM deposition and cytoskeletal ments. Cross-linked actin networks (CLANs), which are mechanosensitive and could affect cellular biomechanical properties,^35^ play an important role in increased AH outflow resistance.^36^^,^^37^ CLANs resembling a geodesic dome-like structure appeared more frequently in the HTM cells exposed to longer durations of hydrostatic pressure (Figure 5D). Western blot results confirmed that the collagen-1, α-SMA, and fibronectin levels gradually increased from the 12 h group to the 1 and 3-day groups (Figure 5E).

### Rap1 pathway was inhibited while YAP1 expression was activated in the TM after acute HIOP

**Figure 6:**
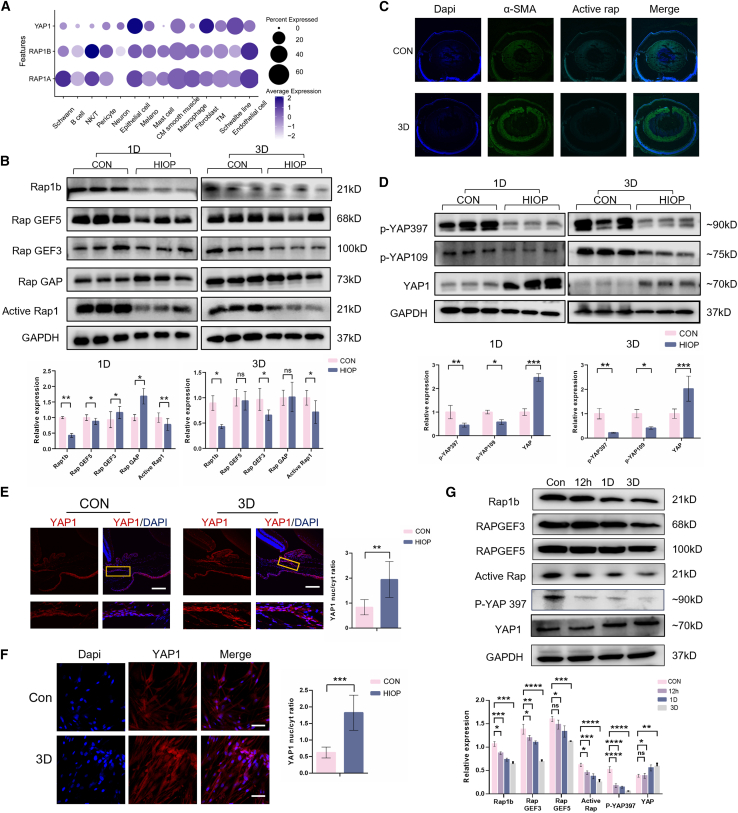
Rap1 pathway was inhibited while YAP1 expression was activated in the TM after acute HIOP (A) Results from single-cell sequencing of human trabecular meshwork (TM) tissues confirm the expression of Rap1a, Rap1b, and YAP1 protein in each cell cluster. (B) Representative western immunoblots showing downregulation of the Rap1 signaling pathway in the TM after anterior chamber perfusion. *N* = 6 mice per group. (C) Immunofluorescence image of the effect of acute intraocular pressure (IOP) elevation on the expression of active Rap1and α-SMA in mice TM tissue. Magnification bars, 250 μm. (D) Relative YAP1 and phosphorylated YAP1 (p-YAP397) levels in the control (CON) and high IOP eyes (HIOP) 1 or 3 days after anterior chamber perfusion, as determined by western blotting. *N* = 6 mice per group. (E) Representative co-immunofluorescence staining of YAP1 (red) and DAPI (blue) in TM tissues, and quantification of the nuclear YAP1 (YAP nuc.) to cytoplasmic YAP1 (YAP cyt.) intensity ratio of cells in TM. Magnification bars: 125 μm. *N* = 6 mice per group. (F) Immunofluorescence of YAP1 (red) and DAPI (blue) in human TM cells under control and high hydrostatic pressure conditions. Pressure treatment induced YAP nuclear accumulation at 3 days. Scale bars, 50 μm. *N* = 6. (G) Western blot analysis of Rap1 and YAP1 signaling pathway activity in HTM cells cultured under control (con) or high hydrostatic pressure for 12 h (12 h), 1 day (1D), and 3 days (3D). *N* = 3. ns, not significant, ∗*p* < 0.05, ∗∗*p* < 0.01, ∗∗∗*p* < 0.001, ∗∗∗∗*p* < 0.0001. Data represent mean ± SD.

The TM underwent cell loss and excessive ECM accumulation after acute HIOP, both of which could contribute to increased AH outflow resistance. However, the underlying mechanisms remain unclear. A previous study indicated that Rap1, a small GTPase, plays a vital role in pathological changes in the TM in glaucoma.^26^ Rap1 was also recently identified as an important mechanosignaling regulator and was therefore investigated herein.^29^^,^^38^ First, we confirmed that Rap1b, Rap1a, and YAP1 were expressed in the HTM using scRNA-seq (Figure 6A). Western blot demonstrated marked decreases in the Rap1b, RAP GEF5, and RAP GEF3 levels and a slight increase in RAPGAP (inhibitor of Rap1b) levels 1 and 3 days after acute IOP elevation, indicating a decrease in Rap1 signaling activation (Figure 6B). Fluorescence immunohistochemistry showed that active Rap fluorescence was lower and α-SMA fluorescence was higher in HIOP eyes than in controls (Figure 6C). To investigate the downstream mechanisms, we examined YAP1, a key contributor to glaucoma pathogenesis and a central mechanotransductive transcription coactivator in the TM.^39^^,^^40^ Western blot analysis revealed that total YAP1 protein increased sharply at 1 and 3 days after anterior segment perfusion in the mouse TM, whereas YAP1 phosphorylation (p-YAP1[S397]) significantly decreased (Figure 6D). To further confirm YAP1 activation, we examined its subcellular localization using fluorescence immunohistochemistry. In the control TM, YAP1 was predominantly localized to the cytoplasm, indicating an inactive state (Figure 6E). Following HIOP, YAP1 showed increased nuclear translocation at 3 and 7 days, with a significant increase in the nuclear/cytoplasmic ratio. Similarly, in HTM cells subjected to high hydrostatic pressure for 3 days, YAP1 translocated from cytoplasm to nucleus robustly (Figure 6F). Therefore, YAP1 signaling was activated following acute HIOP and promoted TM fibrosis.^41^^,^^42^ Western blot analysis of cultured HTM cells also showed that Rap1b expression and activation were inhibited, while YAP1 phosphorylation was suppressed under high hydrostatic pressure (Figure 6G).

### Rap1 influences TM death and ECM disruption by negatively regulating YAP1 signaling following acute HIOP

**Figure 7:**
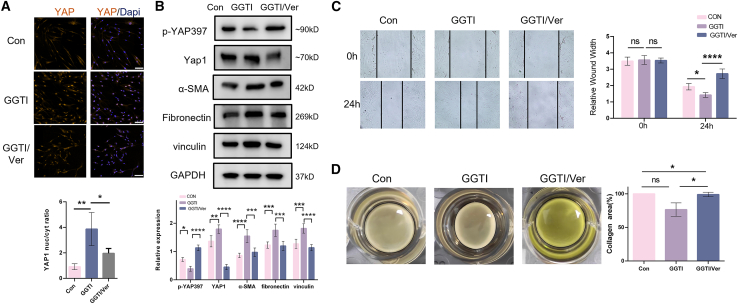
Inhibition of Yap reduced cell migration and contraction in HTM cells (A) Immunofluorescence of YAP1 (red) and DAPI (blue) in human TM cells exposed to high hydrostatic pressure for 24 h, and quantification of the nuclear YAP1 (YAP nuc.) to cytoplasmic YAP1 (YAP cyt.) intensity ratio. Rap1 inhibitor GGTI-298 (GGTI) and YAP1 inhibitor verteporfin (Ver) was used. *N* = 4. (B) Expression of fibronectin, vinculin, α-SMA, YAP1 and phosphorylated YAP1 (p-YAP397) in the different groups of HTM cells by western blots. *N* = 6. (C) Scratch assay of HTM cells treated with or without 10 μM GGTI-298 and 1 μM verteporfin under high hydrostatic pressure for 24 h. The migration inhibition was transformed to the percentage of the initial distance between the two edges. The HTM cells showed a faster rate of wound closure with GGTI-298 treatment than the control cells. *N* = 3. (D) Contraction assay of the HTM-collagen gel complex in the presence or absence of 10 μM GGTI-298 and 1 μM verteporfin under high hydrostatic pressure for 24 h. Statistical analysis of the gel area revealed that verteporfin efficiently inhibited gel contraction induced by GGTI-298. *N* = 3. ns, not significant, ∗*p* < 0.05, ∗∗*p* < 0.01, ∗∗∗*p* < 0.001, ∗∗∗∗*p* < 0.0001. Data represent mean ± SD.

Therefore, Rap1 signaling was significantly reduced following the acute IOP elevation, which might account for the Yap1 activation and TM stiffness. To test this hypothesis, HTM cells were exposed to high hydrostatic pressure (1 kPa) for 24 h while a pharmacological Rap1 inhibitor (GGTI-298) and a YAP inhibitor (verteporfin) was applied. Immunofluorescence staining of Yap1 revealed that 10 μM GGTI-298 promotes Yap1 nuclear localization (Figure 7A), therefore inhibited YAP phosphorylation. Verteporfin (1 μM) reversed robust YAP nuclear localization induced by GGTI-298(Figure 7A). Western blot analysis showed that GGTI-298 treatment resulted in a significant upregulation of total YAP1 protein levels concomitant with a marked reduction in phosphorylated YAP1 (p-YAP1 S397), indicative of enhanced YAP1 transcriptional activation (Figure 7B). Furthermore, GGTI-298 administration substantially increased the expression levels of α-SMA, vinculin, and fibronectin (Figure 7B). Cell scratch tests indicated that YAP1 inhibition significantly suppressed the aberrant cell migration induced by GGTI-298 treatment, further supporting the critical role of YAP1 activation in mediating the cellular phenotypic changes following the inhibition of Rap1 signaling (Figure 7C). Collagen gel contraction assays were performed to assess the contractile activity of HTM cells. GGTI-298 treatment led to significantly lower gel diameter than in controls, indicating markedly enhanced cellular contraction (Figure 7D). Notably, the addition of verteporfin effectively abrogated this excessive contractile response. These findings collectively demonstrate that Rap1 inhibition activates YAP1 nuclear transcriptional signaling and promotes excessive ECM accumulation and cell contraction.

**Figure 8:**
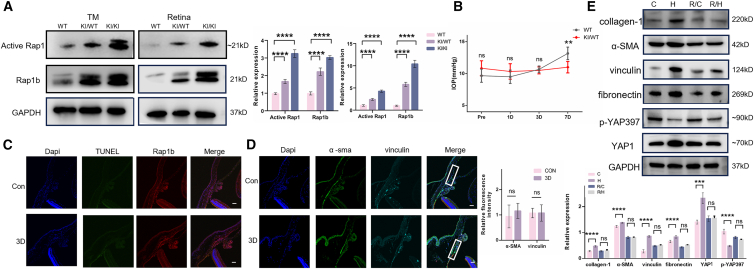
Overexpression of Rap1b alleviated TM damage by negatively regulating YAP1 following acute HIOP (A) The expression of Rap1b and active Rap in the TM and retina from wild-type mice and Rap1b KI mice. WT, wild-type mice; KI/WT, heterozygous knockin mice; KI/KI, homozygote knockin mice. *N* = 6 mice per group. (B) The fluctuation of intraocular pressure (IOP) in the eye of wild-type mice (WT) and heterozygous Rap1b knockin mice (KI/WT) following anterior chamber perfusion. Data represent mean ± SD. Two-way ANOVA revealed no significant differences in IOP between WT and KI/WT eyes after acute IOP elevation across time points, expect for 7 days. ns, not significant, ∗*p* < 0.05, ∗∗*p* < 0.01. *N* = 6 mice per group. (C) Representative images of control and high IOP (HIOP) eyes 7 days after anterior chamber perfusion (7D) immunolabeled for TUNEL. Scale bars, 50 μm. (D) Immunostaining images of heterozygous Rap1b knockin mice 3 days after anterior chamber perfusion. The staining corresponds to the following antibodies: α-SMA (green) and vinculin (cobalt). The nuclei were stained with DAPI (blue). The area marked in the boxes indicates where the TM located. Scale bars, 50 μm. (E) Western blot showing the expression of fibrosis-associated proteins and YAP1. C, control wild-type mice; H, wild-type mice with HIOP; R/C, heterozygous Rap1b knockin mice; R/H, heterozygous Rap1b knockin mice with HIOP. *N* = 6 mice per group. ns, not significant, ∗*p* < 0.05, ∗∗*p* < 0.01, ∗∗∗*p* < 0.001, ∗∗∗∗*p* < 0.0001. Data represent mean ± SD.

Further, Rap1b knockin mice were generated (Figure S1) in which Rap1b was overexpressed and labeled with tdTomato. TM and retinal tissues derived from Rap1b knockin mice demonstrated a significant increase in the Rap1b and active Rap levels (Figure 8A). Eyes from Rap1b knockin mice were perfused at 45 mmHg in the same manner, and the IOP was monitored. Unlike wild-type (WT) mice, Rap1b knockin mice demonstrated no significant increase in IOP 7 days after perfusion (Figure 8B). The TUNEL assay indicated no significant cell death in the AC angle, 7 days following acute HIOP, which was different from in the WT mice (Figure 8C). Fluorescence immunohistochemistry staining and western blot of the TM, both revealed no significant increases in the levels of α-SMA and vinculin (Figures 8D and 8E). Moreover, YAP1 activation was clearly inhibited in Rap1b knockin mice when compared with WT mice after AC cannulation (Figure 8E). Therefore, Rap1b overexpression protected the TM from IOP-induced injury by reducing cell loss and ECM accumulation through suppression of YAP1.

## Discussion

IOP remains the principal and only modifiable risk factor for all forms of glaucoma.^43^ The TM is the most important target for controlling the IOP, as it mediates 80%–90% of the AH outflow. Although elevated IOP is derived primarily from increased outflow resistance at the TM, whether and how HIOP affects the structure and function of the TM remains unclear.

The TM is continuously exposed to mechanical deformation and is regarded as highly mechanosensitive.^19^^,^^20^^,^^44^ However, evidence for the sensitivity of TM cells comes from animal model and cell culture experiments, and these findings must still be verified in humans. The present scRNA-seq analysis suggested enrichment of mechanical response pathways in the TM1 cluster; however, bioinformatic findings alone cannot determine functional mechanosensitivity. Our *in vivo* and *in vitro* experiments examined TM responses at the tissue and cellular levels without subregion-specific resolution but future studies employing subregion-specific isolation techniques are needed to resolve differential susceptibilities across TM subpopulations to guide the development of targeted glaucoma treatments.^45^

The present study provides a detailed characterization of the structural, cellular, and molecular consequences of an acute IOP elevation *in vivo* and the identification of the Rap1-YAP1 axis as a potential therapeutic target. No obvious collapse in the SC lumen was observed following the acute IOP elevation, which is consistent with the observation in living mice visualized by optical coherence tomography that the lumen opens instantly after stabilizing of the IOP from 45 to 10 mm Hg.^46^ Hence, TM dysfunction may be primarily responsible for increased IOP. In the present study, the TM exhibited pronounced cytoskeletal rearrangement, ECM remodeling, and cellular death following IOP elevation. Moreover, the Rap1 pathway was significantly suppressed, whereas both YAP1 expression and its activity were increased. The combined effects of TM cell loss, excessive ECM deposition, and cytoskeletal reorganization likely contribute to increased AH outflow resistance following acute IOP elevation. By preserving TM cell viability and preventing ECM deposition, Rap1b knockin mice preserved the physiological architecture of the outflow pathway, thereby preserving outflow facility.

Our study revealed that acute IOP elevation to 45 mmHg-induced damage to the TM, which appears distinct from transient responses to physiological mechanical stress. Studies have confirmed that cells may recover from certain mechanical load by removing pharmacological treatments or force application.^47^ However, the reversibility of cellular mechanical activation is largely variable depending on the magnitude, directionality, type, and timescales of the mechanical stresses.^48^ While diurnal variations in IOP (typically 2–6 mmHg) are well-tolerated,^49^^,^^50^ acute elevation to 45 mmHg for 60 min might exceed the compensatory capacity of TM mechanosensors and trigger irreversible cell death pathways. Previous studies have shown that acute IOP elevations to 45 mmHg cause significant morphological changes in the outflow pathway, including Schlemm’s canal collapse and meshwork herniations into collector channels in bovine eyes.^51^ Furthermore, a study confirmed that 45 mmHg IOP treatment for 5 min in mice caused significant neurons loss and microglia activation in the retina.^52^ Therefore, the severity of mechanical stress appears critical. While TM cells can recover from mild cyclic stretch or pharmacological perturbations upon treatment cessation, extreme pressure elevation triggers irreversible mechanotransduction cascades that trigger cell death and ECM remodeling, which are inherently associated with permanent tissue changes. This pathological persistence likely reflects the transition from adaptive mechanotransduction responses to maladaptive fibrosis, analogous to the “point of no return” observed in other pressure-related tissue injuries. Moreover, a review introduced a new class of mechanically operated signaling scaffolds that integrate chemical and mechanical signals including activating small GTPase Ras to introduce quantized positional changes to ligands and persistent cytoskeletal architecture alterations which provide “mechanomemory” capabilities.^53^ Sharing similar effector domain and some effector pathways with Ras, Rap1 may also play an important role in providing “mechanomemory” capabilities, which needs to be further investigated.

YAP1 serves as a downstream effector of the conserved Hippo kinase pathway^54^ and has recently been shown to be a nuclear relay for the mechanical stimulation of TM cells.^39^^,^^55^^,^^56^ Stiffened substratum increased the expression and nuclear localization of YAP1 in HTM cells.^57^ During cyclic mechanical stretch, activation of the YAP1 signaling pathway reportedly promoted the disruption of the cytoskeleton and its function and enhanced ECM remodeling in the TM.^58^ Although extensive evidence has confirmed YAP1’s crucial role in the pathological changes in glaucoma,^56^ its upstream regulators remain largely unknown. The present study suggests that Rap1 inhibition following IOP elevation may relieve suppression of YAP1, leading to its nuclear translocation and activation of pro-fibrotic gene programs and actin cytoskeleton reorganization. Rap1 has been established as a key upstream node regulating the activity of the YAP1 signaling pathway but its regulation pattern is highly dependent on cell types and context.^59^^,^^60^^,^^61^ The present data help fill this gap in the understanding of the link between Rap1 and YAP1 signaling, though the precise signaling mechanisms remain to be determined.

In conclusion, building upon the extensive foundation of research on TM mechanosensitivity,^21^^,^^22^^,^^23^ this work extends these findings by focusing specifically on the structural, cellular, and molecular consequences of a defined acute IOP elevation and the potential protective role of Rap1-YAP1 signaling modulation. The Rap1-YAP1 signaling pathway represents a promising and novel therapeutic target for TM protection in glaucoma-associated acute IOP elevation; nevertheless, further clinical investigations are needed.

### Limitations of the study

In our model of acute HIOP, the observed IOP increases 7 days post-injury suggest potentially sustained impairment of the conventional outflow pathway. While our study focused on TM-specific changes, we cannot exclude the potential effects of Rap1 signaling on distal outflow components. Notably, Rap1, expressed in endothelial cells of the SC, has been shown to regulate endothelial barrier function and mechanosensing in lung vascular endothelial cells.^30^^,^^62^ Future studies utilizing segmental perfusion techniques and detailed morphological analysis of the entire outflow pathway will be necessary to determine whether Rap1-mediated protection is specific to the TM or is involved in coordinated regulation of distal outflow tissues. Another limitation of this study is that the selection of the Rap1-YAP1 axis was hypothesis-driven based on our previous work rather than identified through unbiased screening. Future studies employing transcriptomic or proteomic profiling of TM after acute IOP elevation could reveal additional pathways involved in this response. Lastly, the sex and gender might influence the results of the study, which were not explored in our study.

## Resource availability

### Lead contact

Further information and requests for resources and reagents should be directed to and will be fulfilled by the lead contact, Jingyi Zhu (email: dr.zhujingyi@qq.com).

### Materials availability

This study did not generate new unique reagents.

### Data and code availability

• The scRNA-seq can be downloaded from Gene Expression Omnibus (https://www.ncbi.nlm.nih.gov/geo/query) and are publicly available on May 19, 2020. Accession numbers are listed in the key resources table.
• This paper does not report original code.
• The processed data and analysis scripts used in this study are available from the corresponding author upon request.
• Any additional information required to reanalyze the data reported in this paper is available from the lead contact upon request.

## Acknowledgments

This study was financially supported by the 10.13039/501100001809National Natural Science Foundation of China (Grant No. 82201191), 10.13039/501100003819Natural Science Foundation of Hubei Province (Grant No. 2023AFB461), and Key Clinical Specialty in the military-Independent Research Project in Ophthalmology (Grant No. 1821-03).

## Author contributions

Conceptualization, Jingyi Zhu; performed experiments, Yupeng Zhang, Xue Li, Qiumei Hu, and Linlin Luo; data analysis, Yupeng Zhang and Qian Luo; drafting, Jingyi Zhu; editing, Xiangbin Gua and Qian Luo; funding acquisition, Jingyi Zhu.

## Declaration of interests

The authors declare no competing interests.

## STAR★Methods

### Key resources table

**Table:** 

REAGENT or RESOURCE	SOURCE	IDENTIFIER
**Antibodies**		

Anti- Rap1 GTP	NewEast Biosciences	Cat# 26912；RRID: AB_2629414
Anti- fibronectin	Abcam	Cat# ab268020; RRID: AB_2941028
Anti-α-SMA	Cell Signaling Technology	Cat# 19245; RRID: AB_2734735
Anti- vinculin	Bioss Antibodies	Cat# bs-6640; RRID: AB_11048227
Anti- COL1A1	Cell Signaling Technology	Cat# 72026; RRID: AB_2904565
Anti- fibronectin	Abcam	Cat# ab268020; RRID: AB_2941028
Anti- vinculin	Cell Signaling Technology	Cat# 13901; RRID: AB_2728768
Anti- CO-I	Cell Signaling Technology	Cat# 72026; RRID: AB_2904565
Anti- Rap1b	Proteintech	Cat# 10840-1-AP; RRID: AB_2253539
Anti- Rap1 GAP	Abcam	Cat# ab32373; RRID: AB_777621
Anti-Rap GEF5	Invitrogen	Cat# PA5-101811; RRID: AB_2851243
Anti- p-YAP1(S109)	Cell Signaling Technology	Cat# 53749; RRID: AB_2799445
Anti- p-YAP1(S397)	Invitrogen	Cat# PA5-110163; RRID: AB_2855574
Anti- YAP1	Cell Signaling Technology	Cat# 14074; RRID: AB_2650491
Anti- GAPDH	Cell Signaling Technology	Cat# 2118; RRID: AB_561053

**Chemicals, peptides, and recombinant proteins**		

GGTI-298	MedChemExpress	HY-100876
Verteporfin	MedChemExpress	HY-B0146
DAPI	MedChemExpress	28718-91-4
Alexa Fluo 488 Phalloidin	Invitrogen	A12379
Specialty Medium	ScienCell	6591

**Critical commercial assays**		

Active Rap1 Detection Kit	Cell Signaling Technology	Cat# 8818; RRID: AB_3675293
TUNEL kit	Servicebio	G1504
SuperSignal West Femto Maximum Sensitivity Substrate	Thermo Fisher Scientific	34096

**Deposited data**		

Single cell RNA-seq data	van Zyl T et al., 2020^6^	GEO: GSE148371

**Experimental models: Organisms/strains**		

Mouse: C57BL/6J	Beijing Vital River Laboratory Animal Co., Ltd	N/A
Mouse: ROSA26 CAG promoter-mRap1b CDS-P2A-Tdtomato-WPRE-PolyA KI mice	Biocytogen Pharmaceuticals	N/A

**Oligonucleotides**		

Wild-type (WT) allele (469 bp): AGTCGCTCTGAGTTGTTATCAG (forward primer) and TGAGCATGTCTTTAATCTACCTCGATG (reverse primer)	This paper	N/A
KI allele (268 bp): AGTCGCTCTGAGTTGTTATCAG (forward primer) and AGTCCCTATTGGCGTTACTATGG (reverse primer)	This paper	N/A

**Software and algorithms**		

ImageJ software	NIH	https://imagej.nih.gov/ij/
GraphPad Prism (version 8.0.2)	GraphPad Software	https://www.graphpad.com/
R	R project	https://www.r-project.org

### Experimental model and study participant details

#### Animal and cell culture

8–10-week-old C57BL/6J mice of both genders were purchased from Beijing Vital River Laboratory Animal Co., Ltd. (Beijing, China). All the mice used in this study were maintained under pathogen-free conditions on a 12-h light–dark cycle, with free access to food and water. All animal procedures were approved by the Animal Ethics Committee of the General Hospital of Central Theater Command (approval number: 20221016).

HTM cells were acquired from ScienCell and are widely applied in TM research.^15^^,^^63^ The cells were tested for mycoplasma contamination, validated using MGP/CHI3L1/myocilin immunofluorescence and maintained in a complete culture medium supplemented with 10% fetal bovine serum and antibiotics at 37°C and 5% CO_2_. For mimicking ocular hypertension *in vitro*, cells in the HIOP group were cultured under 1 kPa of pressure using a biomimetic pressure cell culture instrument (NK-P40; Shanghai Naturethink Life & Scientific Co., Ltd., Shanghai, China). All experiments used cells between passages three and five.

### Method details

#### Generation and genotyping of ROSA26 CAG promoter-mRap1b CDS-P2A-Tdtomato-WPRE-PolyA KI mice

Rap1b knock-in mice were generated on a C57BL/6J background using CRISPR/Cas9-mediated gene editing. This service was provided by Biocytogen Pharmaceuticals Co. Ltd. (Beijing, China). Single-guide RNAs were designed to target the ROSA26 locus. For the targeting site, candidate guide RNAs were designed using the CRISPR design tool (http://crispr.mit.edu/). Guide RNAs were screened for on-target activity using the Universal CRISPR Activity Assay (Biocytogen Pharmaceuticals, Beijing, China). Cas9 mRNA and sgRNA were transcribed by T7 RNA polymerase *in vitro*. For Cas9 mRNA and sgRNA production, the T7 promoter sequence was added to the Cas9 or sgRNA template using polymerase chain reaction (PCR) amplification. The T7-Cas/sgRNA PCR products were gel purified and used as the template for *in vitro* transcription using the MEGAshortscript T7 kit (AM1354; Thermo Fisher Scientific, Waltham, MA, USA) according to the manufacturer’s protocol. Female C57BL/6J and ICR mice were used as embryo donors and pseudopregnant foster mothers, respectively. Successful KI of the target gene was confirmed at both the genomic DNA level using PCR and the protein level using western blotting.

#### *In vivo* model

Mice were placed under deep anesthesia by intraperitoneal administration of sodium pentobarbital (50 mg/kg), followed by application of 0.05% proparacaine eye drops (Bausch & Lomb, Rochester, NY, USA). Pentobarbital was chosen over ketamine/xylazine because it provides more stable and reproducible anesthetic depth for prolonged procedures (>60 min), as acute elevated IOP model was maintained for 60 min in each mouse.^64^^,^^65^ A 34-G medical-grade needle (Pearle, Jiangsu, China) connected to a sterile saline container (Sigma-Aldrich, St. Louis, MO, USA) was inserted into the AC of one eye. The IOP was increased to 45 mmHg by adjusting the height of the saline reservoir (approximately equivalent to 60 cmH_2_O) for 60 min. In the control group, the AC was cannulated similarly and the IOP was maintained at a normal physiological level of 9 mmHg.

#### Single-cell RNA sequencing data analysis

Data were derived from a previous study^6^ and downloaded from the Gene Expression Omnibus (GSE148371; https://www.ncbi.nlm.nih.gov/geo/query). Six HTM samples and one corneoscleral rim were included in the analysis (GSM4463223, GSM4463229, GSM4463234, GSM4463239, GSM4463244, GSM4463249, and GSM4463253). After quality filtering for the number of detected genes and mitochondrial read counts, the duplicates were removed, and unique transcripts were normalized for the total read depth. Following dimension reduction using principal component analysis performed on the normalized and standardized gene expression matrix using the Seurat R package, the datasets were further integrated across batches using the Harmony algorithm to mitigate the batch effects.^66^ Subsequently, unsupervised clustering was performed to group the cells according to their gene-expression profiles, and t-distributed stochastic neighbor embedding (t-SNE) plots were applied for visualization. Differentially expressed genes (DEGs) between the clusters were used to identify cell types. Gene ontology (GO) enrichment and Kyoto Encyclopedia of Genes and Genomes (KEGG) pathway analyses were conducted using clusterProfiler package.

#### IOP monitoring

IOP was measured in a masked manner using a handheld tonometer (Tonolab; Icare Finland Oy, Vantaa, Finland), with each measurement repeated five times.

#### Transmission electron microscopy

Following dissection, the TM or HTM cells grown on coverslips were immediately immersed in fresh transmission electron microscopy fixative (G1102; Servicebio, Wuhan, China) for 48 h. The samples then underwent post-fixation in a 1% OsO_4_ aqueous solution for 2 h at room temperature. The samples were subsequently washed, dehydrated, embedded in Polybed 812 resin (90529-77-4; SPI Supplies, West Chester, PA, USA), and observed on a transmission electron microscope (HT7800, Hitachi High-Tech, Tokyo, Japan).

#### Histology and fluorescence immunohistochemistry

Eyes were enucleated, fixed in 4% paraformaldehyde, and preserved in 70% ethanol before being processed for paraffin embedding. Samples were sliced at 10 μm and stained using hematoxylin or the TUNEL kit (G1504; Servicebio) according to standard methods. Cultured cells grown on coverslips were fixed and permeabilized, and non-specific binding sites were blocked. Rap1-GTP (26912, 1:100 dilution; NewEast Biosciences, King of Prussia, PA, USA), fibronectin (ab268020, 1:250 dilution; Abcam, Cambridge, UK), α-SMA (19245, 1:250 dilution; Cell Signaling Technology, Danvers, MA, USA), vinculin (bs-6640R, 1:50 dilution; Bioss Antibodies, Woburn, MA, USA), and COL1A1 (72026, 1:250 dilution; Cell Signaling Technology) expression was determined using fluorescence immunohistochemistry. Nuclei were counterstained using DAPI (28718-91-4; MedChemExpress, Monmouth Junction, NJ, USA). All imaging was performed on an Olympus FV3000 microscope (Tokyo, Japan) configured with an argon–krypton laser for fluorescence detection.

#### Western blotting

TM tissue was isolated following previous reports.^67^^,^^68^ The iris, ciliary body, and cornea were gently removed by circular incision under a dissecting microscope (M80; Leica, Wetzlar, Germany). The triangular pigmented TM stripe in the area of the scleral spur was gently isolated using fine forceps. Specifically, each western blot lane pooled TM tissue from 6 mice. Pooled samples were stored at −80°C after flash freezing in liquid nitrogen. Mouse TM or HTM cells were lysed on ice using radioimmunoprecipitation assay buffer for 10 min at 4°C. Following centrifugation at 12,000 rpm for 15 min at 4°C, the supernatant was collected, mixed with loading buffer, and boiled. Protein samples were resolved using sodium dodecyl sulfate-polyacrylamide gel electrophoresis and transferred to polyvinylidene difluoride membranes (03010040001; Roche, Basel, Switzerland). Immunoblotting was performed using specific antibodies against fibronectin (ab268020, 1:1000 dilution; Abcam), α-SMA (19245, 1:1000 dilution; Cell Signaling Technology), vinculin (13901, 1:3000 dilution; Cell Signaling Technology), CO-I (72026, 1:1000 dilution; Cell Signaling Technology), Rap1b (10840-1-AP, 1:1000 dilution; Proteintech, Rosemont, IL, USA), p-YAP1(S397) (PA5-110163, 1:1000 dilution; Invitrogen, Carlsbad, CA, USA), and YAP1 (14074, 1:1000 dilution; Cell Signaling Technology), with GAPDH (2118, 1:1000 dilution; Cell Signaling Technology) serving as a loading control. Active Rap1 was detected using the Active Rap1 Detection Kit (8818; Cell Signaling Technology). After incubation with horseradish peroxidase-labeled secondary antibodies at room temperature for 1 h, immunoreactive bands were revealed using SuperSignal West Femto Maximum Sensitivity Substrate (34096; Thermo Fisher Scientific).

### Quantification and statistical analysis

Data are reported as mean ± standard deviation. All data were processed and analyzed in GraphPad Prism. Western blot bands were measured using ImageJ software (http://ImageJ.nih.gov/ij/; provided in the public domain by the National Institutes of Health, Bethesda, MD, USA). The Student’s *t* test was applied for statistical analysis between the two groups. For a comparison of more than two group, analysis of variance was used. Precise details about experimental group size are indicated in the figure legends. Significance was set at *p* < 0.05. ∗*p* < 0.05, ∗∗*p* < 0.01, ∗∗∗*p* < 0.001, ∗∗∗∗*p* < 0.0001.
