# Supplementary material for: Targeting Rap1-YAP1 mechanosignaling for ameliorating acute IOP elevation-induced trabecular meshwork dysfunction
Source: iScience. 2026 Jun 5;29(6):116268. doi: 10.1016/j.isci.2026.116268 (PMC13264258; doi:10.1016/j.isci.2026.116268)

iScience, Volume *29*

Supplemental information

Targeting Rap1-YAP1 mechanosignaling for ameliorating acute IOP elevation-induced trabecular meshwork dysfunction

Yupeng Zhang, Xue Li, Qiumei Hu, Linlin Luo, Qian Luo, Xiangbin Guan, and Jingyi Zhu

Figure S1. Generation and genotyping of ROSA26 CAG promoter-mRap1b CDS-P2A-Tdtomato-WPRE-PolyA KI mice.

A. Targeting strategy of ROSA26 CAG Promoter-mRap1b CDS-P2A-Tdtomato-WPRE-PolyA KI mice.

B. Genotype identification of wild-type (WT), heterozygous (KI/WT), and homozygous (KI/KI) mice. A female F1 heterozygous mouse was used as a positive control (PC).

C. Photographs of littermate postnatal 7-day wild-type (WT), heterozygous (KI/WT), and homozygous (KI/KI) mice. The skin appeared red in heterozygous and homozygous mice and dark in wild-type mice.

D. Brain, heart, kidney, and liver of adult wild type (WT), heterozygous (KI/WT), and homozygous (KI/KI) mice.

E. Images of the anterior segments of adult wild-type (WT), heterozygous (KI/WT), and homozygote (KI/KI) mice under a slit lamp. No significant differences were observed in the cornea, iris, anterior chamber, or lens between the groups. However, the red reflex was intense in both heterozygous and homozygous mice because of the presence of red retinas.

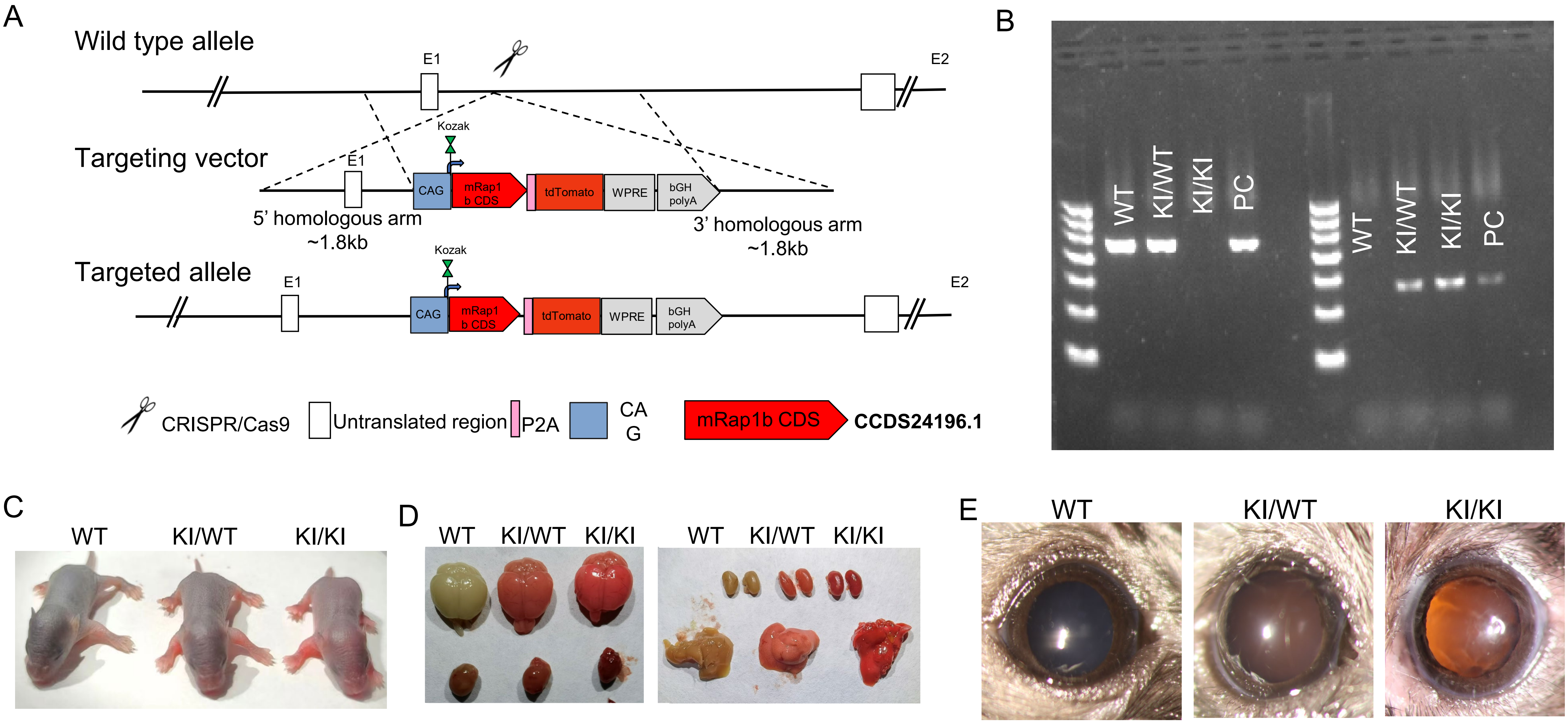
A
Wild type allele
E1
E2
Targeting vector
Kozak
E1
CAG
mRap1 b CDS
tdTomato
WPRE
bGH polyA
5' homologous arm ~1.8kb
3' homologous arm ~1.8kb
Targeted allele
Kozak
E1
E2
CAG
mRap1 b CDS
tdTomato
WPRE
bGH polyA
CRISPR/Cas9
Untranslated region
P2A
CA G
mRap1b CDS
CCDS24196.1
B
WT
KI/WT
KI/KI
PC
WT
KI/WT
KI/KI
PC
C
WT
KI/WT
KI/KI
D
WT
KI/WT
KI/KI
WT
KI/WT
KI/KI
E
WT
KI/WT
KI/KI

Figure S2. Western blotting validation of mice TM tissue.
Western blotting validation was performed using trabecular meshwork-specific markers aquaporin-1(AQP1), Matrix gla protein (MGP), chitinase-3 like-1(CHI3L1). Negative controls sample were extracted from the retina.

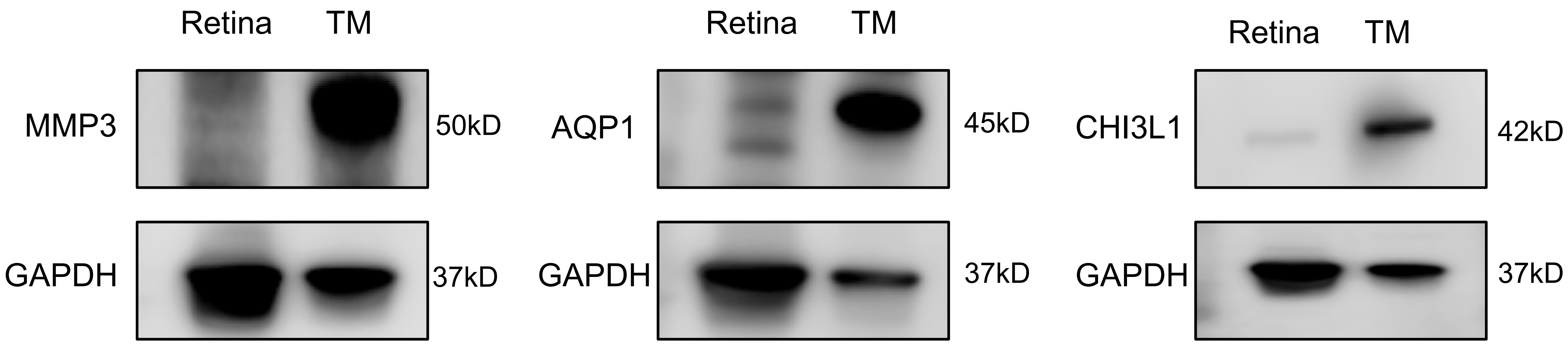

Supplement: Document S1. Figures S1 and S2 [file mmc1.pdf]
